# Extended repertoire of CXC chemokines acting as agonists and antagonists of the human and murine atypical chemokine receptor ACKR2

**DOI:** 10.1093/jleuko/qiaf013

**Published:** 2025-02-04

**Authors:** Rafael Luís, Brian F Volkman, Martyna Szpakowska, Andy Chevigné

**Affiliations:** Immuno-Pharmacology and Interactomics, Department of Infection and Immunity, Luxembourg Institute of Health (LIH), 29, rue Henri Koch, L-4354 Esch-sur-Alzette, Luxembourg; Faculty of Science, Technology and Medicine, University of Luxembourg, 2, place de l'Université, L-4365 Esch-sur-Alzette, Luxembourg; Department of Biochemistry, Medical College of Wisconsin, Milwaukee, WI 53226, United States; Immuno-Pharmacology and Interactomics, Department of Infection and Immunity, Luxembourg Institute of Health (LIH), 29, rue Henri Koch, L-4354 Esch-sur-Alzette, Luxembourg; Immuno-Pharmacology and Interactomics, Department of Infection and Immunity, Luxembourg Institute of Health (LIH), 29, rue Henri Koch, L-4354 Esch-sur-Alzette, Luxembourg

**Keywords:** ACKR3, CXCL1, CXCL10, CXCL11, CXCL12, CXCL2, CXCL5, CXCR3, d6

## Abstract

Atypical chemokine receptors are a subfamily of important regulators of chemokine functions. Among them, ACKR2 has long been considered a scavenger of multiple inflammatory chemokines exclusively from the CC family. Recently, we demonstrated the ability of ACKR2 to scavenge the CXC chemokine CXCL10, previously reported to bind solely the classical receptor CXCR3. This discovery emphasized the need for systematic reassessments of chemokine–receptor pairings. In this work, we established a highly sensitive NanoBRET-based competition binding assay using a novel proprietary ACKR2 modulator (LIH222) and applied it in a comprehensive reassessment of the pairings between human and murine chemokines and their respective ACKR2 orthologs. We confirmed CXCL10 as a ligand for the human but also the mouse ACKR2. We also identified CXCL5, CXCL11, and CXCL12 as new CXC chemokines for both ACKR2 orthologs. Furthermore, we showed that CXCL2 is a ligand for the human but not the mouse ACKR2, whereas CXCL1 binds the mouse but not the human receptor. Finally, we found that N-terminally truncated CXCL5 (CXCL5_8-78_) loses its capacity to bind ACKR2, whereas the removal of the first 2 residues of CXCL11 (CXCL11_3-73_) enhances its antagonist potency, showing a tendency toward a reduction of the receptor basal interactions with β-arrestins. Altogether, this study demonstrates that ACKR2 is not exclusive to CC chemokines, and although with a weaker affinity, it can also bind and scavenge a subset of inflammatory and homeostatic CXC chemokines important for the regulation of the immune system.

## Teaser

A novel NanoBRET-based competition binding assay using fluorescent LIH222 for a systematic reassessment of chemokine–receptor pairings reveals several CXC chemokines as new ligands for the human and mouse ACKR2.

## 1. Introduction

Atypical chemokine receptors (ACKRs) do not trigger G protein signaling and cell migration upon chemokine binding, in contrast to the classical chemokine receptors. Nevertheless, ACKRs play an important role in the chemokine network, acting as decoy or scavenger receptors regulating the availability of the ligands in the extracellular environment and therefore controlling the activation of classical chemokine receptors.^1–4^

ACKR2, formerly known as D6 or CCBP2, is an important scavenger for a broad range of chemokines. ACKR2 is expressed mostly on lymphatic endothelial cells, trophoblasts, and some subsets of immune cells such as innate-like B cells and alveolar macrophages.^5–7^ Both murine and human ACKR2 were discovered in 1997, with these studies simultaneously revealing the high promiscuity of both orthologs for inflammatory chemokines of the CC family exclusively.^8–10^ ACKR2 was shown to bind CCL2–8, CCL11–14, CCL17, and CCL22, which are ligands of the classical CC receptors CCR1–5.^8–12^ The pairing of ACKR2 with CC chemokines dates back to when some chemokines were not yet discovered or readily available,^13–15^ hindering the complete profiling of the chemokine binding repertoires. This was verified more than 2 decades later when CXCL10, previously reported to solely bind CXCR3, was identified as a potent partial agonist of the human ACKR2 ortholog.^16^ This unexpected ability of ACKR2 to interact with chemokines from both CC and CXC families highlighted the gaps in our understanding of chemokine network and leukocyte biology and the need for a systematic reassessment of chemokine–receptor pairing to explore the potential remaining interactions.

In this study, we took on the challenge of determining the full chemokine repertoire of ACKR2 and providing new insights of potential roles of this receptor in the physiological and pathological contexts. Previous studies elucidating the pairings of atypical chemokine receptors have relied mainly on using radiolabeled chemokines.^10^ Alternative assays like those monitoring β-arrestin recruitment to the receptor, relying on Nanoluciferase (NanoLuc) complementation (NanoBiT) or bioluminescence resonance energy transfer (BRET),^16–18^ have been developed in the past years. Although they allowed for important breakthroughs, it remains a challenge to systematically assess the activity of chemokines and to identify weak agonist or antagonist chemokines. Furthermore, intrinsic differences between human and murine orthologs, including in their interactions with transducers, and the lack of unbiased probes for their simultaneous investigation represent additional obstacles.

## 2. Methods

### 2.1 Chemokines

Native chemokines were purchased from Protein Foundry. CXCL5_8-78_ was purchased from PeproTech, and CXCL11 mutants were previously described.^19^ Cy5-labeled chemokines were generated using Cy5 N-hydroxysuccinimide ester QuickStain protein labeling kit (Amersham) according to the manufacturer's protocol.

### 2.2 Cell lines

HEK293T cells (Abcam) were grown in Dulbecco's modified Eagle medium (Gibco) supplemented with 10% fetal bovine serum (Sigma) and penicillin/streptomycin (100 U/mL and 100 µg/mL, respectively; Gibco). HEK-ACKR2 cell lines stably expressing human or mouse ACKR2 N-terminally fused with the NanoLuc (hACKR2 and mACKR2, respectively) were established using the pIRES-puromycin vector and selected using 5 µg/mL of puromycin (InvivoGen).

### 2.3 β-Arrestin recruitment

Ligand-induced β-arrestin recruitment to receptors was monitored by NanoBRET as previously described.^17,20^ Briefly, 6 × 10^6^ HEK293T cells were plated in 10-cm dishes and cultured for 24 h before cotransfection with vectors encoding chemokine receptors C-terminally tagged with mNeonGreen and β-arrestin-1 N-terminally tagged with the NanoLuc. Twenty-four hours after transfection, cells were harvested and distributed into white 96-well plates (1 × 10^5^ cells per well), and chemokines were added at the indicated concentrations. Subsequently, coelenterazine H was added to the wells. Donor emission (450/8 nm bandpass filter) and acceptor emission (600 nm longpass filter) were measured with a GloMax Discover microplate reader (Promega). Results shown correspond to average values acquired for 30 min, represented as a percentage of maximum full agonist response.

### 2.4 Ligand-binding competition monitored by NanoBRET

Ligand binding to ACKR2 was monitored by NanoBRET. HEK293T cells stably expressing the receptor N-terminally fused to NanoLuc were distributed into white 96-well plates (5 × 10^4^ cells per well). Increasing concentrations of ligands were added to the cells, as well as LIH222_AZ594_ (10 nM), and incubated for 2 h on ice. Coelenterazine H was then added, and donor emission (450/8 nm BP filter) and acceptor emission (600 nm LP filter) were immediately measured on a GloMax Discover plate reader (Promega). BRET binding signal was defined as acceptor/donor ratio, and cells not treated with LIH222_AZ594_ were used to define 0% binding, whereas cells that were treated with LIH222_AZ594_ alone were used to define 100% binding.

### 2.5 Binding and internalization of Cy5-labeled chemokines

HEK293T cells stably expressing human or murine ACKR2 (2.5 × 10^5^) were incubated for 1 h at 37 °C or 4 °C in the presence of 100 nM Cy5-labeled chemokines. Cells were washed twice with flow cytometry staining buffer (PBS, 1% BSA, 0.1% NaN_3_ sodium azide) and subjected or not to proteinase K treatment (0.1 mg/mL) for 3 h at 4 °C to evaluate chemokine binding to the cell surface. Cells were washed twice with FACS buffer and incubated for 20 min at 4 °C with Zombie Green viability dye (BioLegend). After 2 phosphate-buffered saline washes, the fluorescent chemokine binding and uptake was quantified by mean fluorescence intensity on a BD LSRFortessa cytometer (BD Biosciences).

## 3. Results and discussion

With a novel proprietary modulator LIH222, recognizing both human and mouse ACKR2 with a strong affinity, we monitored ligand–receptor interactions in competition binding studies using a highly sensitive approach based on NanoLuc bioluminescence resonance energy transfer (NanoBRET). In this assay, the receptor is N-terminally fused with the NanoLuc, and upon catalysis of the substrate, the luminescence generated is transferred to the fluorophore coupled with LIH222. Importantly, neither the receptor fusion nor the probe labeling affects the interactions between chemokines and the receptor. Moreover, in this assay, only the ligand interaction with the NanoLuc-fused receptor is monitored, allowing the exclusion of the contribution of glycosaminoglycans, which is an advantage over approaches relying on radiolabeled ligand binding.

**Fig. 1.**
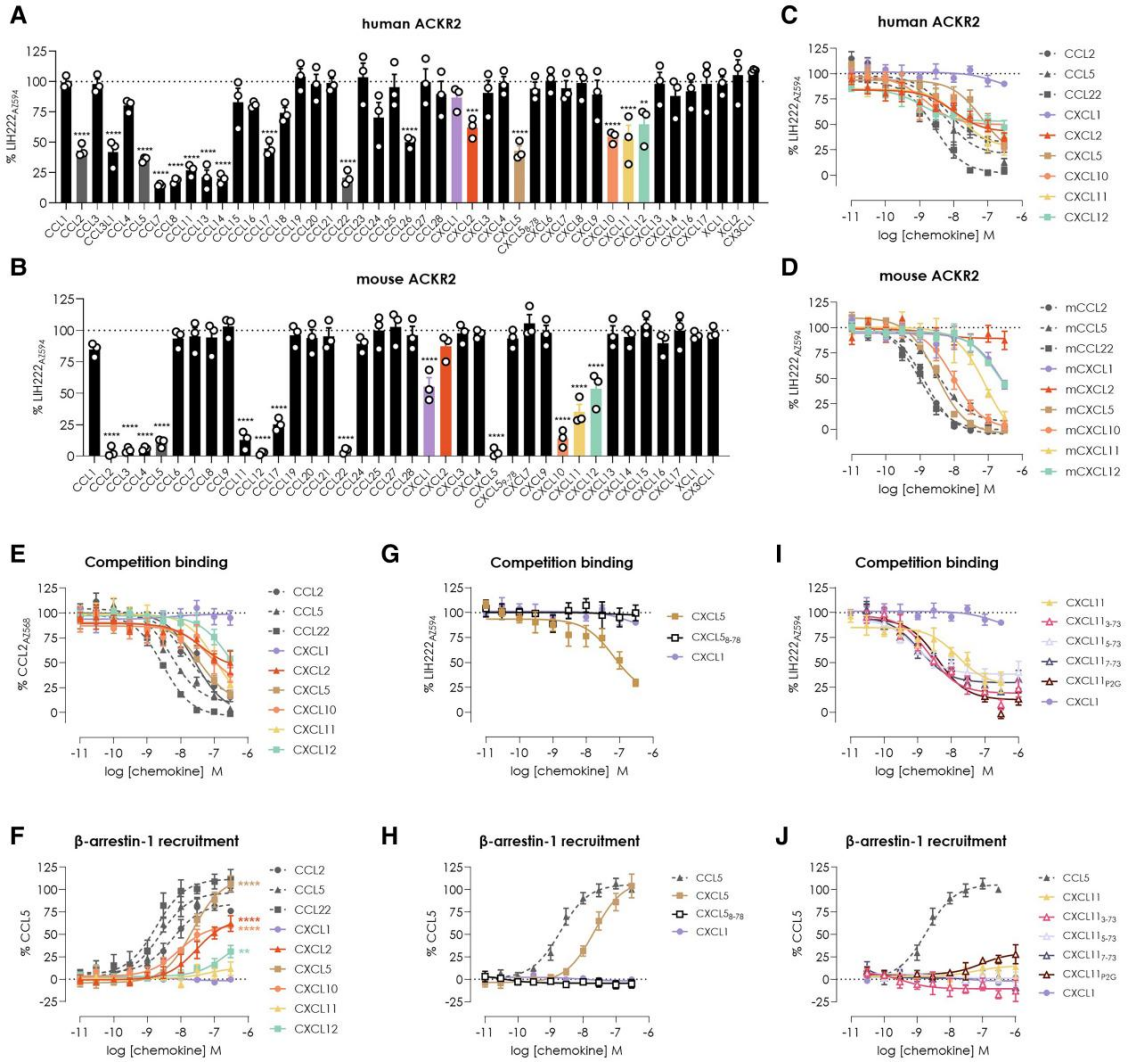
Systematic reassessment of human and mouse ACKR2 chemokine repertoire. A, B) Competition binding screening of all known human (A) and mouse (B) chemokines on the respective ACKR2 orthologs determined by NanoBRET. HEK293T cells expressing NanoLuc-ACKR2 were incubated with AZDye594-labeled LIH222 (LIH222-AZ594) and competitor chemokines (100 nM) for 2 h on ice. LIH222-AZ594 concentration was determined based on the EC_80_ values (5 nM for human ACKR2 and 10 nM for mouse ACKR2). C, D, E, G, I) Concentration–response curves of selected ligands from the CXC family in competition binding with LIH222-AZ594 (5 nM for human and 10 nM for mouse) or AZDye568-labeled CCL2 (CCL2-AZ568) (10 nM) determined by NanoBRET on HEK293T cells expressing either human (C, E, G, I) or mouse (D) ACKR2 N-terminally tagged with NanoLuc. Human and mouse CCL2, CCL22, and/or CCL5 were used as positive controls. F, H, J) Concentration–response curves of selected ligands from the CXC family monitored by NanoBRET in β-arrestin-1 recruitment to ACKR2. Data represented as a percentage of maximal response obtained with the full agonist CCL5 (300 nM). For statistical analysis, 1-way analysis of variance (ANOVA) (A, B) or 2-way ANOVA (F, H, J) with Bonferroni's multiple comparison was performed. *P < 0.05, **P < 0.01, ***P < 0.001, ****P < 0.0001. In panel F, statistical significance is shown for the highest concentration tested. All chemokines, except CXCL11, showed statistically significant differences compared to CXCL1, which was used as a negative control. Data are represented as mean ± SEM of at least 3 independent experiments (*n* = 3).

We first screened all the chemokines at a concentration of 100 nM for their ability to displace LIH222 from ACKR2. In agreement with previous reports, our results confirmed that both human and murine ACKR2 bind a broad range of CC inflammatory chemokines,^9–11^ attesting to the accuracy of the NanoBRET-based technique (Fig. 1A and 1B). Human ACKR2 strongly interacted with CCL2, CCL3L1, CCL4, CCL5, CCL7, CCL8, CCL11, CCL13, CCL14, CCL17, and CCL22 (Fig. 1A and Supplementary Fig. 1). The screening also confirmed our previous report that CCL26 is an additional CC chemokine ligand for human ACKR2 (Fig. 1A).^21^ Remarkably, CXCL10 was not the only CXC chemokine identified for ACKR2. CXCL2, CXCL5, CXCL11, and CXCL12, which are ligands for CXCR1 and CXCR2, CXCR3, and CXCR4, respectively, showed significant displacement of LIH222 from ACKR2. For mouse ACKR2, strong binding was observed with CCL2, CCL3, CCL4, CCL5, CCL11, CCL12, CCL17, and CCL22 but also several CXC chemokines, including CXCL1, CXCL5, CXCL10, CXCL11, and CXCL12, showing that the ability of ACKR2 to interact with chemokines from the 2 families is shared between the human and the mouse receptors (Fig. 1B).

Next, using the same assay, we investigated the concentration–response relationship of the newly identified human and mouse chemokines and compared the potencies of both ACKR2 orthologs. For human ACKR2, we observed that CXCL2, CXCL5, CXCL10, CXCL11, and CXCL12 displaced LIH222 with similar potencies and reduced the BRET signal by 50% to 70% at the highest concentration tested (300 nM), similar to what we observed with the CC chemokine CCL2 (Fig. 1C and Supplementary Table 1). Interestingly, for mouse ACKR2, a slightly different profile from that of the human ortholog was observed. While CXCL5 (pIC_50_ ≤ 8.51), CXCL10 (pIC_50_ ≤ 8.01), and CXCL11 (pIC_50_ ≤ 7.04) were the strongest binders among the CXC ligands and displaced almost completely LIH222 at the highest concentration tested, CXCL1 and CXCL12 showed overlapping profiles, displaying partial displacement of the probe (∼50%) (Fig. 1D and Supplementary Table 1). Moreover, while CXCL2 was shown to be a ligand for the human but not the mouse ACKR2, CXCL1 displayed an opposite behavior, binding the mouse but not the human receptor.

While human CXCL2 and CXCL12 were already suggested as ACKR2 agonists in our previous screening using β-arrestin recruitment as the readout,^16^ the interactions between CXCL5 and CXCL11 with ACKR2 were only identified in the present study using competition binding. To rule out the use of our new ACKR2 probe as accountable for this discrepancy, we first compared the competition binding results obtained with LIH222_AZ594_ to those generated with an endogenous ACKR2 chemokine, CCL2, labeled with a similar dye (CCL2_AZ568_). The displacements observed with labeled CCL2 confirmed CXCL2, CXCL5, CXCL10, CXCL11, and CXCL12 as ligands of human ACKR2, with slightly reduced potencies compared to those measured with LIH222 (Fig. 1E and Supplementary Table 1). We then evaluated the agonist activity of all these chemokines in a β-arrestin recruitment assay (Fig. 1F). Interestingly, CXCL5, but not its truncated variant (CXCL5_8-78_), used in our previous study,^16^ was able to displace LIH222 and acted as full agonist (Fig. 1G and 1H). CXCL11 and CXCL12 appeared as weak ACKR2 agonists, with CXCL11 barely able to induce β-arrestin recruitment, suggesting, in light of its strong binding to the receptor, an antagonist activity (Fig. 1I). Furthermore, we found that P2G mutation (CXCL11_P2G_) or the removal of the first 2 residues of CXCL11 (CXCL11_3-73_), mimicking the N-terminal cleavage by the dipeptidyl peptidase 4 (DPP4 or CD26), both reported to turn the chemokine from CXCR3 agonist to antagonist,^19^ had a different effect on ACKR2. P2G mutation appeared to slightly increase the efficacy of the chemokine to induce β-arrestin recruitment (pIC_50_ = ≈ 7.25 ± 0.35, Emax = 28%), while the removal of the first 2 residues, although the statistical significance was not reached, seemed to turn it into an inverse agonist capable of inhibiting the constitutive interaction of ACKR2 with β-arrestin (Emax = −17%) (Fig. 1J).

**Fig. 2.**
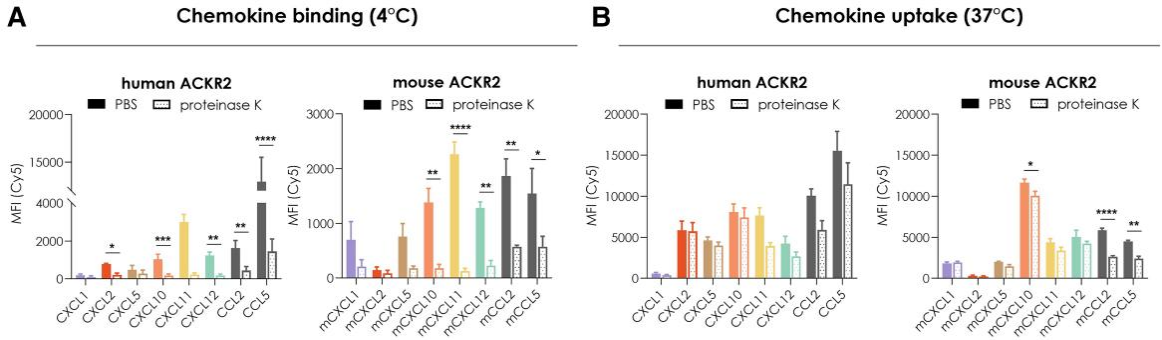
Binding and internalization of CXC chemokines by human and mouse ACKR2. A, B) Cell surface binding (4 °C) (A) and intracellular accumulation (37 °C) (B) of Cy5-labeled CXCL1, CXCL2, CXCL5, CXCL10, CXCL11, and CXCL12 (100 nM) monitored by flow cytometry. Results are expressed as mean fluorescence intensity (MFI). Data shown are presented as mean ± SEM of 3 independent experiments. HEK293T cells expressing either human or mouse ACKR2 were incubated with the corresponding species-specific chemokines for 1 h. Subsequently, cell surface–bound chemokines were removed by proteinase K treatment. CCL2 and CCL5 were used as positive controls. For statistical analysis, 2-way ANOVA with Bonferroni's multiple comparison test was performed. *P < 0.05, **P < 0.01, ***P < 0.001, ****P < 0.0001.

ACKR2 being established as a scavenger receptor for CC chemokines, we then monitored its ability to bind and mediate the intracellular uptake of the newly identified CXC chemokines using flow cytometry and ligands coupled with the Cyanine5 (Cy5) dye. The binding assay performed at 4 °C confirmed that all CXC chemokines were able to bind to ACKR2-expressing cells (Fig. 2A) but not the untransfected cells (Supplementary Fig. 2). This binding could be abolished by the treatment with proteinase K, a nonselective protease allowing the removal of cell surface–bound chemokines. In contrast, except for human CXCL11, this treatment had no impact on the signal detected following incubation at 37 °C, conditions allowing internalization, therefore strongly pointing to intracellular uptake of the CXC chemokines upon receptor binding (Fig. 2B and Supplementary Fig. 2).

**Fig. 3.**
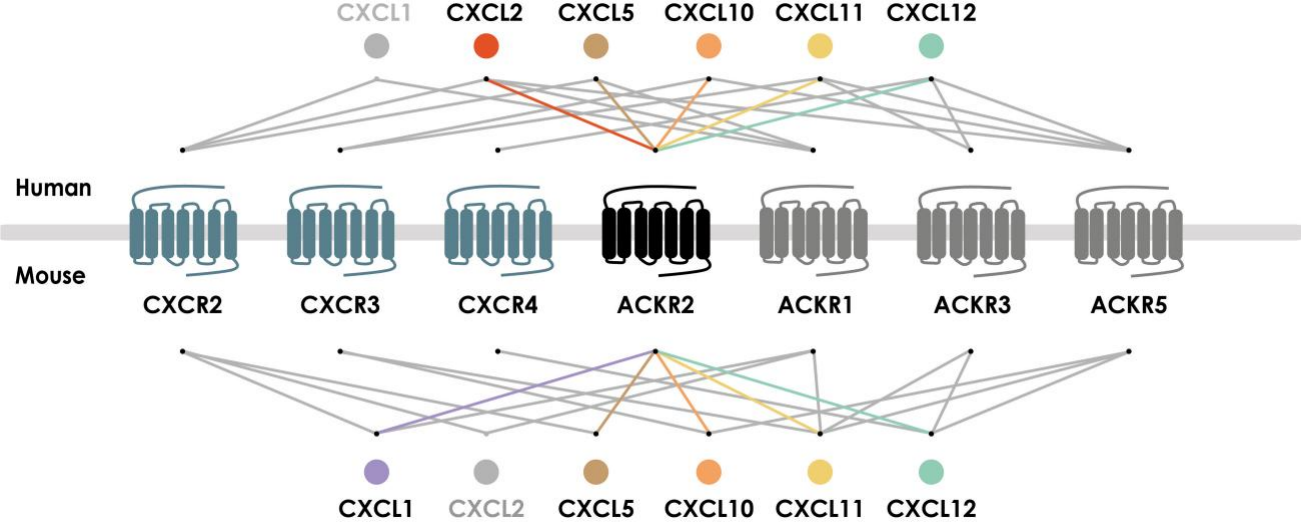
CXC chemokine network of ACKR2. Schematic representation of the chemokine pairings of human (top) and mouse (bottom) ACKR2, including other atypical and classical chemokine receptors described to bind these CXC chemokines.

Altogether, this systematic screening program confirmed both ACKR2 orthologs as receptors for a broad range of known CC chemokines but also the previous pairing of ACKR2 with CXCL10.^16,21^ Additionally, it allowed the identification of CXCL5, CXCL11, and CXCL12 as ligands for both human and murine ACKR2. The interactions between human CXCL2 and ACKR2 and between mouse CXCL1 and ACKR2 were also pinpointed for the first time, increasing the complexity of the involvement of ACKR2 within the chemokine network and highlighting once more the differences between the human and the murine chemokine–receptor networks (Fig. 3). This study also identified CXCL11 as the first antagonist chemokine of ACKR2 and demonstrated the importance of chemokine N-terminal processing for chemokine scavenging and the negative modulation of basal activity of the receptor.

These findings indicate that ACKR2 may exert an extended regulatory role through the scavenging of certain CXC chemokines and the control of their availability and signaling through their classical receptors, notably CXCR1/CXCR2, CXCR3, and CXCR4. In particular, the 2 inflammatory chemokines CXCL10 and CXCL11, both signaling through CXCR3, were identified as the strongest CXC binders of ACKR2, showing divergent (i.e. agonist vs antagonist) activity, emphasizing the possible functional interconnection between these 2 receptors and their ligands, especially during inflammatory processes. ACKR2 interactions with CXCL1/CXCL2/CXCL12 may have additional relevance as these chemokines are implicated in the maturation of certain subsets of hematopoietic cells, and ACKR2 has been shown to be present in hematopoietic cell progenitors.^6^ Interestingly, most of these chemokines are already known to bind to other ACKRs,^22–24^ suggesting that the interaction between these ligands and the respective ACKRs is context- and compartment-dependent or that a synergism between ACKRs exists, as recently reported.^25^

All these new pairings will require complementary pharmacological and functional experimental confirmation beyond β-arrestin recruitment. Notably, several reports point to increased CXC chemokine levels, such as CXCL10, in ACKR2-deficient mice.^26,27^ Overall, this study underscores once again the need for a comprehensive and systematic reevaluation of pairings with native chemokines but also with forms resulting from proteolytic processing for both well-established and newly identified receptors. The relevance of altered receptor selectivity and affinity of differentially processed chemokines should be further established also in light of the previously reported cell type–dependent presence of such forms and the possible related spatiotemporal regulation of the chemokine network.^28,29^ Lastly, this report also highlights the ability of ACKR2 to scavenge chemokines from both CC and CXC families, which seems to represent an additional property shared by most ACKRs.

## Supplementary Material

qiaf013_Supplementary_Data

## Data Availability

All data are available upon reasonable request.

## Abbreviations

ACKR: atypical chemokine receptor
BRET: bioluminescence resonance energy transfer
CCL: chemokine CC motif ligand
CCR: chemokine CC motif receptor
CXCL: chemokine CXC motif ligand
CXCR: chemokine CXC motif receptor
NanoBiT: NanoLuc Binary Technology
