# Supplementary material for: Extended repertoire of CXC chemokines acting as agonists and antagonists of the human and murine atypical chemokine receptor ACKR2
Source: J Leukoc Biol. 2025 Feb 4;117(4):qiaf013. doi: 10.1093/jleuko/qiaf013 (PMC12017343; doi:10.1093/jleuko/qiaf013)
Supplement: qiaf013_Supplementary_Data [file qiaf013_supplementary_data.zip › Supplementary Table 131224.docx]

|  | **Competition Binding** | | | | | | | | |
| --- | --- | --- | --- | --- | --- | --- | --- | --- | --- |
|  | **CCL2** | **CCL5** | **CCL22** | **CXCL1** | **CXCL2** | **CXCL5** | **CXCL10** | **CXCL11** | **CXCL12** |
| **Dmax (%)**  **(LIH222-AZ594)** | 73 | 87 | 96 | N.D. | 62 | 70 | 64 | 70 | 53 |
| **Dmax (%)**  **(CCL2-AZ568)** | 85 | 96 | 100 | N.D. | 50 | 85 | 62 | 73 | 46 |

**Human**

|  | **β-arrestin-1 Recruitment** | | | | | | | | |
| --- | --- | --- | --- | --- | --- | --- | --- | --- | --- |
|  | **CCL2** | **CCL5** | **CCL22** | **CXCL1** | **CXCL2** | **CXCL5** | **CXCL10** | **CXCL11** | **CXCL12** |
| **pEC_50_** | 8.20 ± 0.12 | 8.59 ± 0.10 | 8.74 ± 0.11 | N.D. | 7.53 ± 0.18 | 7.64 ± 0.10 | 8.08 ± 0.13 | N.D. | ≈ 7.03 ± 0.02 |
| **Emax** | 76 | 100 | 112 | N.D. | 62 | 106 | 59 | 12 | 31 |

**Mouse**

|  | **Competition Binding** | | | | | | | | |
| --- | --- | --- | --- | --- | --- | --- | --- | --- | --- |
|  | **CCL2** | **CCL5** | **CCL22** | **CXCL1** | **CXCL2** | **CXCL5** | **CXCL10** | **CXCL11** | **CXCL12** |
| **pIC_50_**  **(LIH222-AZ594)** | ≥ 8.87 | ≥ 8.45 | ≥ 9.02 | N.D. | N.D. | ≥ 8.51 | ≥ 8.01 | ≥ 7.04 | N.D. |
| **Dmax (%)**  **(LIH222-AZ594)** | 100 | 95 | 100 | 56 | N.D. | 99 | 96 | 88 | 56 |

**Table 1.** Competition binding data. Dmax: percentage of displacement of labaled chemokines (LIH222-AZ594 or CCL2-AZ594) at the highest concentration tested, Emax: percentage of β-arrestin-1 recruitment to ACKR2 at the highest concentration tested with respect to response obtained with the full agonist CCL5 (300 nM), N.D.: not determined. Values are presented as mean ± SEM of three independent experiments.
