# Supplementary figures and images for: Extended repertoire of CXC chemokines acting as agonists and antagonists of the human and murine atypical chemokine receptor ACKR2
Source: J Leukoc Biol. 2025 Feb 4;117(4):qiaf013. doi: 10.1093/jleuko/qiaf013 (PMC12017343; doi:10.1093/jleuko/qiaf013)

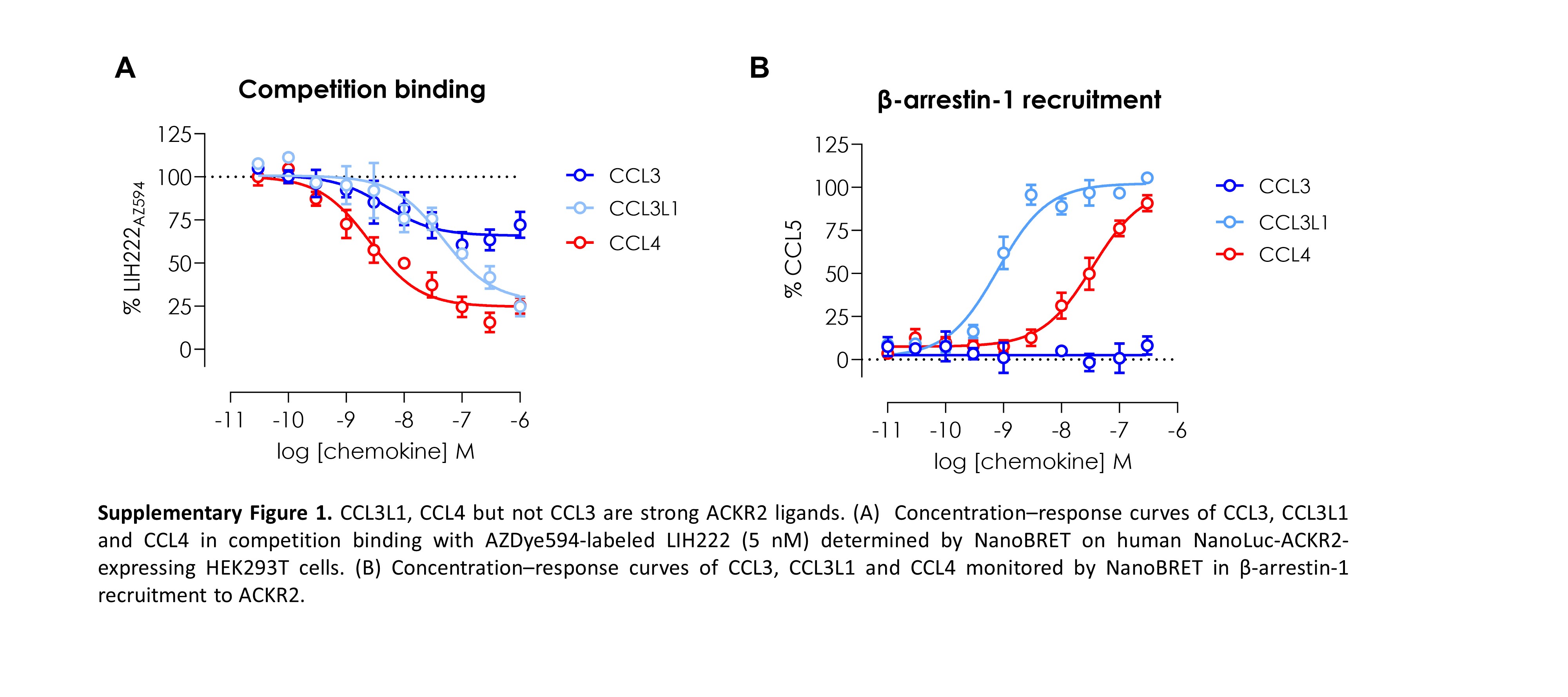

Supplement: qiaf013_Supplementary_Data [file qiaf013_supplementary_data.zip › Supplementary Figure 1.jpg]

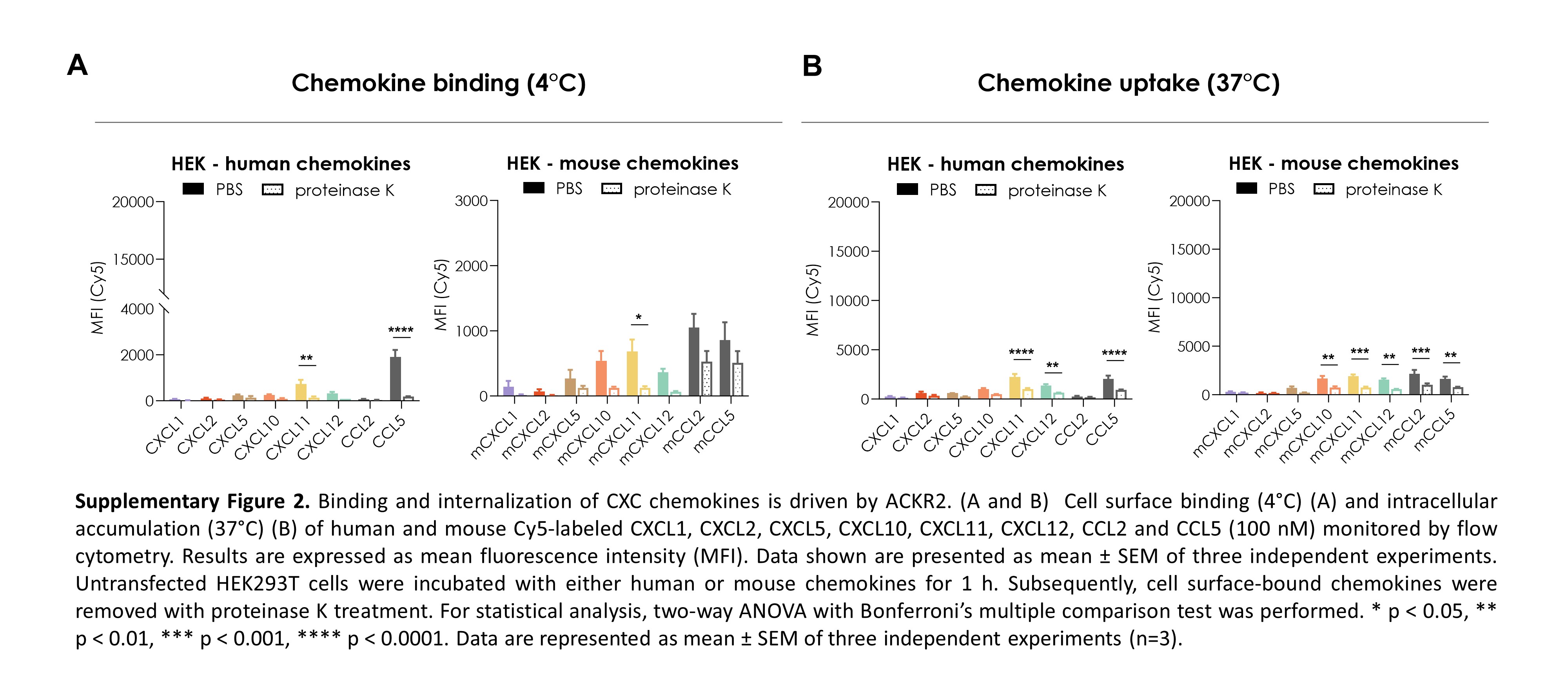

Supplement: qiaf013_Supplementary_Data [file qiaf013_supplementary_data.zip › Supplementary Figure 2.jpg]
